# Parental food provisioning is related to nestling stress response in wild great tit nestlings: implications for the development of personality

**DOI:** 10.1186/1742-9994-12-S1-S10

**Published:** 2015-08-24

**Authors:** Kees van Oers, Gregory M Kohn, Camilla A Hinde, Marc Naguib

**Affiliations:** 1 Department of Animal Ecology, Netherlands Institute of Ecology (NIOO-KNAW), Wageningen, The Netherlands; 2 Behavioural Ecology Group, Department of Animal Sciences, Wageningen University, Wageningen, The Netherlands; 3 current address: Department of Psychological and Brain Sciences, Indiana University, Bloomington IN, USA

**Keywords:** Animal personality, Early development, Parental feeding, Food type, Stress response, Great tit, Taurine, Spiders, Caterpillars

## Abstract

**Background:**

Variation in early nutrition is known to play an important role in shaping the behavioural development of individuals. Parental prey selection may have long-lasting behavioural influences. In birds foraging on arthropods, for instance, the specific prey types, e.g. spiders and caterpillars, matter as they have different levels of taurine which may have an effect on personality development. Here we investigated how naturally occurring variation in the amounts of spiders and caterpillars, provisioned to nestlings at day 4 and 8 after hatching, is related to the response to handling stress in a wild passerine, the great tit (*Parus major*). Broods were cross-fostered in a split-brood design allowing us to separate maternal and genetic effects from early rearing effects. Adult provisioning behaviour was monitored on day four and day eight after hatching using video recordings. Individual nestlings were subjected to a handling stress test at an age of 14 days, which is a validated proxy for exploratory behaviour as an adult.

**Results:**

Variation in handling stress was mainly determined by the rearing environment. We show that, contrary to our predictions, not the amount of spider biomass, but the amount of caterpillar biomass delivered per nestling significantly affected individual performance in the stress test. Chicks provisioned with lower amounts of caterpillars exhibited a stronger stress response, reflecting faster exploratory behaviour later on in life, than individuals who received larger amounts of caterpillars.

**Conclusions:**

These results suggest that natural variation in parental behaviour in wild birds modulates the developmental trajectories of their offspring's personality via food provisioning. Since parental provisioning behaviour might also reflect the local environmental conditions, provisioning behaviour may influence how nestlings respond to these local environmental conditions.

## Introduction

An important period in the life of an animal is the developmental period before independence. In many organisms it has now been shown that the environment during this early life stage can have significant and long-term effects on behaviour later on in life [1-3] as well as the next generation [4,5]. Moreover, recent studies show that personality related traits such as exploration behaviour [6,7] or handling stress [8] are affected by the conditions experienced during early development. Consistent behavioural variation among individuals, also referred to as personality, is thereby biologically meaningful and can have potential evolutionary consequences [9-11]. Plasticity in personality traits seems to counteract the basic assumption of personality being an individual constraint on behavioural flexibility. Yet, numerous studies have shown that many traits identified as personality traits can change over an individual's lifespan [12]. Moreover, individuals have been found to consistently differ in their plasticity [13]. Studies in birds [14-17], mammals [18], invertebrates [19,20] and fish [21,22] have shown that individuals also consistently differ in how plastic they are in their responsiveness to environmental conditions, especially when individuals are in conditions in which they must compete with others.

Thus, the development of personality traits has been shown to be under both genetic and environmental control [23,24]. Personality traits have a moderate to high heritability [23,25] suggesting that non-genetic factors can also substantially contribute to the expression and development of personality traits [26,27]. Environmental conditions that associate with variation in competition, such as food availability, might actively influence the development of personality traits, such that individuals will be more adapted to their adult environment [28]. The relative proportion of genetic, epigenetic, parental and environmental factors in the development of the phenotype has thus become of interest in evolutionary and behavioural biology.

The environment during the early period in life is highly dependent on the parents. These so-called parental effects occur when the phenotype of the parents or the environment they experience affects the phenotype of their offspring [e.g. 29]. Parental effects are considered “common” environmental effects that offspring experience. Maternal hormones, for example, transferred to the eggs and embryos can influence both gene expression and phenotypic expression later in life [30-32]. Parental effects may also consist of modulation of environmental conditions in a process often called niche construction [33]. This can be most clearly seen in nest building in which parents actively construct and maintain a stable environment for the early development of their offspring [see e.g. 34]. Few studies in natural populations have specifically tested how parental feeding behaviour during dependence affects the development of animal personality traits [but see 35, 36]. The influences of early parental effects are especially apparent in altricial species where parents directly mediate the nutritional, social and environmental conditions their offspring face during early development [37].

The quantity and quality of nutrition is a factor which the parents may vary when providing care to their offspring, especially during the main growth period. Parental prey selection, for instance, will determine not only the quantity of food but also the quality, such as the provisioning of specific nutrients required during early development [38,39]. One of such nutrients, taurine, an essential sulfonated amino acid, is a ubiquitous compound found in almost all animal tissues and is crucial for the functional development of nearly all vertebrates (but especially so in birds and mammals; [40]). Taurine has a modulating influence on the nervous system, and specifically it can act as a neuro-inhibitor of the hypothalamic-pituitary-adrenal (HPA) system and thus have cascading influences into endocrine responses. Studies in rats have shown that the HPA system plays a strong role in regulating the stress response of animals, and that administering a taurine antagonist in this area causes a stronger stress response to environmental challenges [41]. Taurine has also been shown to stimulate cognitive performance [35] and feelings of well-being in humans [42] and also could be important in the development of personality traits by influencing development of the nervous system. Most mammals possess the ability to synthesize their own taurine from closely related amino acids (such as cysteine and methionine). Birds, on the contrary, lack the ability to synthesize most amino acids, and thus must rely on food sources for adequate supplementation. Ramsay and Houston [43] studied the amino-acid composition of woodland arthropods that are common prey items of passerines. They found that spiders contained 40-100 times more taurine than other arthropod food sources. It has previously been suggested that spiders arean essential part of the diet in Parids, and studies have recorded a high proportion of spiders in the early nestling's diet irrespective of season [e.g. 44, 45-47]. To identify the effects of taurine on development of behaviour, Arnold et al.[35] conducted a supplementation experiment in which taurine was added to the diet of nestling wild blue tits (Cyanistescaeruleus). In an experiment, wild blue tit broods were supplemented with 0.5 milligrams of taurine every five days from 2-14 days after hatching. When the nestlings were 15 days old they were brought into the laboratory and subsequently tested for spatial learning and novel object tasks. The authors found that taurine supplementation during early development had cascading behavioural effects later on in life, such as lower neophobia, and increased risk taking behaviours compared to control broods. Based on these findings, we hypothesized that parents can use the amount of spider biomass delivered to the nest as a source of taurine to adjust the subsequent behaviour of their offspring.

Here we identify early parental factors influencing a personality trait in a wild population of great tits. We specifically tested for the effects of parental care and food type by monitoring cross-fostered split-broods with parents of known personality types and recording the food delivered to own and cross-fostered nestlings. Cross-fostering experiments break the co-variation between the rearing environment and the genetic parents during development [48]. By determining the amount of spider biomass, which contains a relatively large amount of taurine, and caterpillar biomass we aim to detect the relation between prey type and the response to handling stress, a validated pre-fledging measure predicting exploratory behaviour at independence [49]. We specifically aimed at assessing whether 1) the handling stress was mainly determined by heritable or by rearing factors, and 2) the amount of spider biomass delivered to the nest associates with the expression of handling stress as a predictor for exploratory behaviour later on in life. We expected that birds who receive more taurine during early development, will show higher breath rates at day 14, which has been shown to associate with faster exploring, and more risk taking individuals [49].

## Results

As expected we found that the weight of nestlings on day two, at the moment of cross-fostering, was determined by the nest of origin and not by the nest of rearing (Table 1). Of the variation in weight at cross-fostering, 50% could be attributed to the nest of origin. At day eight after hatching, so six days after cross-fostering half of the nestlings to other nests, this origin effect decreased to a non-significant 13% of the variation. In contrary, nest of rearing now explained 60% of the variation in weight. After being in a foster nest for 12 days (age 14 days), none of the variation in weight could be attributed to the nest of origin and 51% was due to effects of the rearing environment (Table 1). These results show that the cross-foster experiment successfully separated nest of origin effects from rearing effects and that the influence of the rearing environment in determining weight increased after cross-fostering. The variation in handling stress response was mainly due to variation in the rearing environment (49%), while hardly any variation could be attributed to the nest of origin (0.2%), indicating the large scope for early developmental effects on this predictor for exploratory behaviour.

**Table 1:** Variation in weight measurements explained by nest of origin and nest of rearing environment.

	Nest of Origin				Nest of Rearing				Residual			
Variable	Variance	Wald Z	P	Prop	Variance	Wald Z	P	Prop	Variance	Wald Z	P	Prop
Weight d2	**0.61**	**1.99**	**0.04**	**0.51**	0.007	0.10	0.95	0.00	**0.586**	**6.34**	**<0.001**	**0.49**
Weight d8	0.68	1.64	0.10	0.13	**3.12**	**2.19**	**0.03**	**0.60**	**1.30**	**6.32**	**<0.001**	**0.25**
Weight D14	0.00*	NA	NA	0.00	**1.11**	**2.35**	**0.02**	**0.51**	**1.05**	**6.72**	**<0.001**	**0.49**
Breath rate	0.02	0.31	0.75	0.02	**0.56**	**2.21**	**0.03**	**0.48**	**0.58**	**6.38**	**<0.001**	**0.50**

A linear mixed model was used with nest of origin and nest of rearing as random factors, both nested within cross-foster pair.

Significant effects are shown in bold.

*Component is redundant.

**Figure 1:**
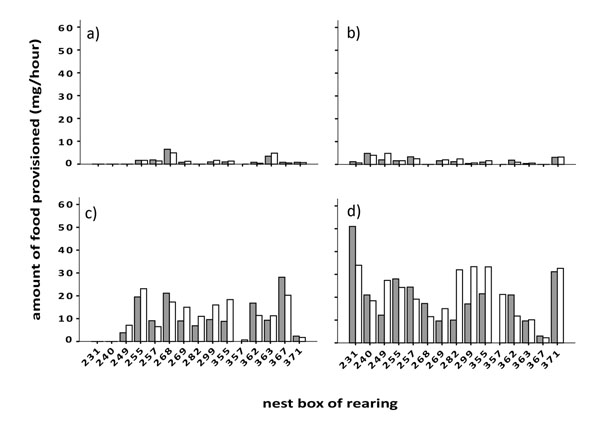
Grey shaded bars representing the original chicks and the white bars the cross-fostered chicks reared in the same nest. With (a) representing the spider biomass on day four, (b) the spider biomass on day eight, (c) the caterpillar biomass on day four and (d) the caterpillar biomass on day eight.

**Figure 2:**
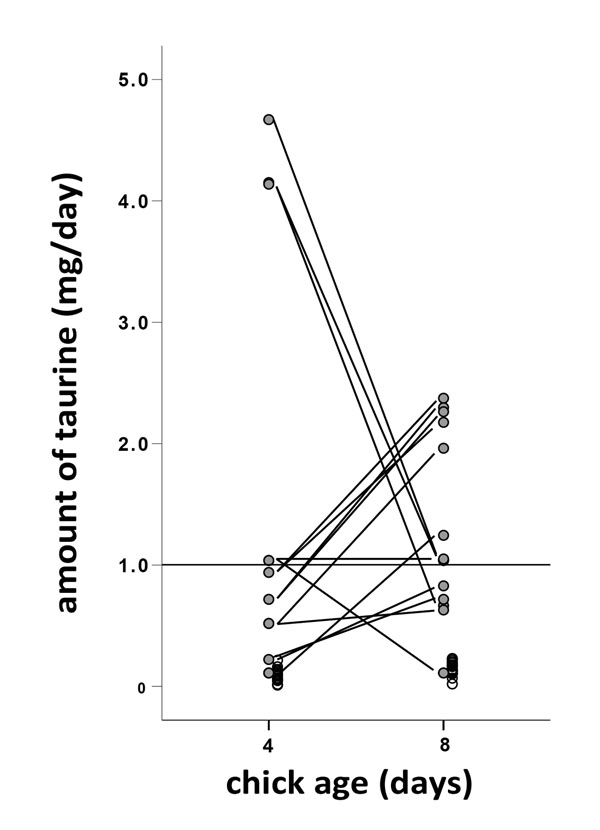
Spiders are indicated with shaded symbols and caterpillars with open symbols. For the amount of taurine delivered to the nests, via spiders, the nests are connected with lines. The horizontal line indicates the amount of taurine supplemented to blue tit (Cyanistescaeruleus) nestlings in the study of Arnold et al. [35].

Furthermore, we found that there was large variability in the amount of spider biomass and caterpillar biomass delivered per nestling on day four compared to day eight (Figure 1). On day four similar amounts of spider biomass were delivered to the nest compared to what was delivered on day eight. Although more caterpillar biomass was delivered on day eight compared to day four, there was no difference in the proportion of spiders between days four and eight (t1,24 = -1.19, P = 0.24). From this biomass of spiders and caterpillars we calculated the amount of taurine that was provisioned to individual chicks. In general spiders contributed most to the amount of taurine a chick received (F1,20.3 = 25.6, P< 0.001; Figure 2). Most of the nests received between 0.1 and 1.2 mg/day of taurine per nest, which was mainly due to spiders. However, both on day four and on day eight, some nests received a considerably higher amount of taurine, up till about 4.7 mg/day (Figure 2). Moreover, many nests did not differ in the amount of taurine between day four and day eight, indicating that there were considerable individual differences. Overall there was only a tendency that chicks received more taurine on day four compared to day eight (F1,23.2 = 4.03, P< 0.06).

**Figure 3:**
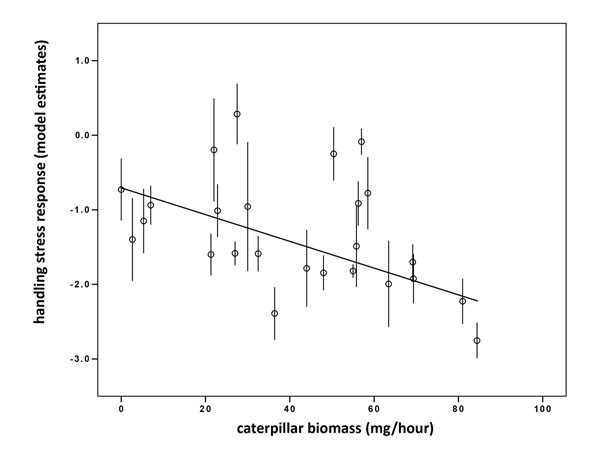
Shown are data for day four with nest means for both original and cross-fostered nestlings within a nest of rearing.

We subsequently investigated how the variation in food supply of the rearing parents on day four and eight affected the response to handling stress of the individual chicks at day 14. Contrary to our expectations we did not find that the amount of spider biomass on day four or eight affected the handling stress response (Table 2). However, the handling stress response on day 14 was affected by the amount of caterpillar biomass delivered to the nest. This was most evident for the amount of caterpillar biomass delivered on day four (Figure 3) where a higher amount of caterpillar biomass on day four resulted in a higher decrease in breath rate at day 14, hence a stronger stress response. The amount of caterpillar biomass on day eight showed a trend in the same direction, however, only when it was in the model together with caterpillar biomass on day four (Table 3). The weight on day 14 did not associate with the handling stress response (Table 3). Moreover, adding weight to the handling stress model did not change the outcome of the effects of caterpillar biomass on day four or eight, indicating that the effect of food provisioning was not mediated via weight differences between the nestlings.

**Table 2:** The effect of food type and amount on breath rate.

Food Type	Age chicks	DF	F	P
spiders	Day 4	1,14.8	0.05	0.82
	Day 8	1,17.4	1.00	0.33
caterpillars	Day 4	1,16.4	6.28	0.02
	Day 8	1,18.6	3.60	0.07
weight	Day 14	1,103	0.47	0.50

A linear mixed model was used with nest of origin and nest of rearing as random factors, both nested within cross-foster pair.

**Table 3:** General information from the handling stress test.

Period	Bout	Mean	S.E.M.	Mean slope	S.E.
1	1	27.9	.36	-1.06	0.09
	2	26.6	.34		
	3	25.2	.34		
	4	24.9	.36		
2	1	28.4	.32	-1.31	0.14
	2	27.3	.31		
	3	25.3	.29		
	4	25.7	.38		

See text for detail

## Discussion

Here we show by using a partial cross-fostering experiment that the early rearing environment had a profound effect on the handling stress response of nestling great tits on day 14, which is a predictor for exploratory behaviour in great tits[49]. Against our predictions, this effect did not operate via the amount of taurine the parents delivered during the first days after hatching, but via the amount of caterpillar biomass on day four, and to a lesser extent that of day eight. These results are partly in line with a study that food deprived whole broods of great tits originating from lines bi-directionally selected for exploratory behaviour[7]. There, growth rates decreased and food-deprived slow explorers became faster compared to control broods, while broods from pairs originating from the line selected for fast exploration, did not react to the experimental food deprivation. Interestingly our results show an independence of the amount of food they get overall.

Our findings that early food provisioning affects personality traits later in life expand on a previous study by Arnold et al.[35] who artificially increased the amount of taurine in blue tit nestling diets and showed that juveniles that were taurine-supplemented as nestlings performed better in spatial learning and novel object exploration tasks. The amount of dietary taurine may still be an important environmental influence on the developmental of behaviour and parental provisioning behaviour thus might have implications for the development of behavioural phenotypes. We, however, found that the amount of taurine supplied via spiders was not greater on day four after hatching than on day eight after hatching, which is not consistent with previous findings [46,50]. Moreover, the variance on day four was large and there was no consistent increase in many nests from day four to day eight. Also the lack of an effect of taurine on the variation in handling stress in this study shows that the relationship between taurine and behaviour may not be as straightforward in natural situations.

The cross-fostering procedure successfully broke the co-variation between the rearing environment and common origin. We found that about half of the variation in weight at cross-fostering was due to nest of origin effects, but that this influence diminished after being in a foster nest for seven days. The weight 14 days after the cross-foster experiment was mainly explained by variation in the nest of rearing. Moreover, when partitioning the variation in handling stress, we found that most of this variation was attributable to the rearing environment. This finding expands on earlier studies that pointed towards a nest of origin effect, since individuals from lines selected for exploratory behaviour differed in levels of handling stress [49] and handling stress was found to have a heritable component [51]. By controlling for the genetic component, we here show that parental food provisioning may be a strong candidate for such a structural environmental component. Permanent environmental effects, which affect all offspring from one nest in the same way, might therefore be important in several personality traits [52,53]. Recent work on the correlational structure of the stress response showed that baseline and stress-induced glucocorticoid concentrations correlated within individuals due to an environmental co-factor, but were not repeatable per se [54]. Other studies in birds have also shown that food deprivation during early development increase rates of stress later on in life [55,56]. A cross-foster study in zebra finches found that these early behavioural differences extend to behaviour during adulthood, indicating that these changes in behaviour might have long-lasting effects [57].

The difference in the proportion of spiders between day four and eight, and subsequently the amount of taurine chicks received via spiders at early age was not as evident as was found in other studies [46,50]. This could have multiple explanations. First of all, most studies that found a correlation between the proportion of spiders in the diet and age were conducted on Parid species other than great tits. The degree to which individuals are selective for prey types could therefore be species specific, and less prevalent in great tits. Some indications for this were also found in a study that investigated the seasonal effects of food availability in great tits [58]. They found that the variation in spider abundance in the diet was mainly due to seasonal changes in availability and selection, but not due to chick age. However, this study only considered chicks aged 7-12 days, while the effect might be higher at younger ages [46,50]. Moreover, we found great variety in the increase or decrease in the proportion of spiders provisioned between nests, with some nests showing high selectivity at day four, while others showed no age effect. Taking only a small number of nests, which could be a limitation to our study, could therefore lead to a biased view [see e.g. 46]. Another reason might be the individual differences in prey type availability and choice itself. Spiders and caterpillars may not be equally distributed across space and some territories might be richer in some prey types than others. Thus unless parents respond to local prey availability by longer search times and more distant foraging locations, they may be constrained in optimizing the balance of prey types. Moreover, individuals can differ in prey selection and in finding local prey and these differences could be personality dependent [58,16]. Unfortunately we did not have enough breeding pairs of which we had exploration scores of both parents, so that we could not explore this further. Yet from other studies it is known that individual pair members consistently differ in their prey choice [e.g. 59] and that parents are affected by the personality of their partner [60]. An interesting approach would for example be, to investigate whether seasonal effects affect the expression of personality traits via seasonal variation in laying date [61] and in food supply [60,55]. Food types may vary differently within and between seasons [50,62,58,63], which may well lead to adaptive responses in preparation for a particular environment.

Fluctuating environments may result in the evolution of alternative developmental trajectories, which seek to maximize survival and fitness according to the current conditions without compromising responsiveness to future conditions. Fluctuating selection can result in differential survival and fitness of individuals with different personality traits. Our study suggests that, if a change in personality expression is adaptive, birds should benefit from becoming faster explorers in situations of low food availability. However, earlier studies have found that aggressive fast explorers are not more successful in situations where there is high competition [64]. We also found handling stress variation to be independent from the weight of the nestlings. In our population, fitness data of three years revealed that faster, bolder females survived better in poor winters compared to slower, shyer females, but in males the pattern was opposite [65]. More interestingly, recruitment rates showed more stabilizing selection patterns in poor years, but this effect was apparent for females only and not for males. Experiments are needed that specifically aim to alter the phenotypes of birds in natural situations and follow them in order to measure the fitness consequences. An interesting example of this would be to actively supplement nests in the wild with both extra spiders and caterpillars at varying degrees and determine the consequences for offspring behaviour. This could help to identify developmental trade-offs and conflicts between food type and the production of bold and shy personality types and the subsequent fitness consequences.

Studies in both wild and captive birds have shown that good conditions during early development can result in,for example, an increased clutch size later in life [66], body mass and size [67,68], amount of subcutaneous fat during migration[69], and quality of breeding and territorial habitat the birds later occupy [70]. The development of personality traits could share some mechanistic similarities with that of other phenomena such as imprinting and song learning, because of the role of early developmental experiences in shaping later behavioural outcomes and their influence on fitness [8]. While this study does not specifically address the neural correlates of personality, it does suggest some important environmental and parental factors that could influence the generation of different neural structures and networks that implicate personality traits. Often the first steps to discovering sensitive or critical periods are to observe the plasticity of behaviour in individuals and conditions experienced during development.

## Conclusion

Plasticity (or environmental responsiveness) and development (the phenotypic changes that integrate both environmental and genetic inputs during an individual's lifetime) are ubiquitous qualities of living beings. In order to understand the maintenance of adaptive variation in personality it is necessary to include the mechanisms through which this variation arises. This study suggests that the amount of caterpillar food provisioned to the nestlings during the first few days after hatching had a larger influence on the expression of a personality trait than variation in the number of spiders delivered, when controlling for the genetic background. Since spiders contain an increased level of taurine, this suggests that the amount of food has a larger effect compared to the amount of taurine. Our results imply that food type may differentially influence developmental trajectories, but that parents could more easily modulate the behavioural phenotype of the offspring by manipulating the amount of caterpillars delivered during early nestling phases, than aiming for a specific food type. This effect is present irrespective of the weight of the nestlings, suggesting an effect on the early growth rather than on the fledging condition. In conclusion, this suggests that parents may be able to passively or actively modify the phenotype of their offspring via food availability during early development.

## Methods

### Subjects and study site

We conducted the study in April and May 2008 using a nest-box population of great tits at the Warnsborn estate near Arnhem, The Netherlands. The study site consists of mixed pine-deciduous woodlands and grassy meadows, and contains a continually monitored population of great tits and about 150 nest boxes that are regularly distributed. Great tits typically lay a clutch of five to 12 eggs that hatch after an incubation period of 13-14 days and nestlings fledge from 16 to 23 days after hatching. From the first week of April onwards we checked nest boxes weekly to determine which boxes were in use. The great tit has been a model system for the study of animal personality, with long-term studies in both wild and captive populations [14]. This experiment was approved by the Institutional Animal Care and Use Committee: the Koninklijke Nederlandse Akademie van Wetenschappen – Dier Experimenten Commissie (KNAW-DEC license NIOO 07.05 to KVO).

### Cross-fostering

When nest building was completed, nest boxes were monitored 3 times a week to record when individuals started laying eggs and started incubation. This allowed us to estimate the expected hatch dates for each brood. Nests with expected hatch dates within a day of each other were assigned to a cross-foster pair. We cross-fostered chicks among broods only when the parental exploratory scores(see below) of both pair members from each brood was known (N = 24 nests). This meant we had 12 pair-combinations between which we cross-fostered offspring. One week before the expected hatch date all nest boxes were monitored daily. We employed a partial cross-foster design where half of the nestlings were swapped between nests and the other half stayed in their nest of origin. On day two after hatching nestlings were weighed and ranked according to their weight. Half of the nestlings in each nest of a cross-foster pair were assigned to be swapped based on their weight rank, with the even or odd ranks being randomly assigned to be staying in the nest or being cross-fostered. In this way cross-fostered chicks did not differ in weight from chicks that stayed in the nest (LMM with nest of origin as random factor; F1,98.4 = 0.06, P = 0.81). Nestlings possess a series of down tufts on their head, arm wing and back that were selectively plucked to identify individuals and their nest of origin. To be able to identify nestlings after cross-fostering each individual was plucked in such a way that nest of origin, as well as their individual identity could be recognized. Down codes were used to identify individuals up until about day seven to nine after hatching, after which individuals were provided with numbered aluminium bands (Vogeltrekstation, Netherlands). On days 2, 8 and 14 after hatching all birds were weighed in order to deduce individual growth patterns. Due to the desertion or predation of nine nests, we have full data of 15 rearing nests (105 offspring) for our analysis. Mean brood size was 7.0 (range 4 to 11) and there were just as many offspring that were cross-fostered (3.8 ± 0.3) compared to offspring that stayed with their natural parents (3.4 ±0.3).

### Video monitoring and analysis

On days four and eight after hatching we recorded the provisioning behaviour of the parents using a CCD 420TV infrared spy-camera installed in the lid of the nest box. It was used to record activity for 2 hours and 20 minutes (maximum battery life) on an Archos 604 multimedia player (Archos Benelux). Since there are daily fluctuations in feeding effort of the parents, all videos were set up between 09:00 and 12:00 pm. In order to be identified during video analyses, cross-fostered nestlings received Tipp-ex marking on their heads and backs, before filming. Videos were analysed to assess parental food provisioning behaviour using Jwatcher Video V1.09 program (http://www.jwatcher.ucla.edu/). The shortest recording was 1 hour and 10 minutes. For this reason we analysed all videos for 50 minutes, to standardize between broods. We started the analysis 20 minutes after the recorders were switched on to make sure the parents were feeding at normal rates. During video analysis we collected data on the type of food; i.e. spider, caterpillar, fly (diptera) or true bug (hymenoptera). The size of food was estimated using the individual's culmen (a straight line from the tip of the beak to where the feathering starts). We categorized the food into 5 different size categories (1-5), with 1 representing half of the culmen, 2 one culmen length, 3 two, 4 three and 5 four culmen lengths. Half a culmen size represents 0.01g of caterpillar fresh weight. We used the formula 0.1269 x fresh weight^1.128 for Operophtera from Bullock and Smith [71], to calculate the dry weight (DW) of the size classes of both caterpillars and spiders brought to either the foster or original chicks. This was then transformed into a measure of mg per nestling per hour by correcting for the observation time and brood size. To assess the amounts of taurine present in the caterpillars and spiders, we used the mean amino acid composition for these arthropod samples (late May caterpillars and wolfspiders in Table 2 from [43]). The identity of the parent was determined using sex specific plumage patterns and (colour) rings.

### Handling stress test

On day 14 the birds were subjected to a handling stress test described in detail in [49]. In brief, the nestlings were removed from the nest box and all placed in a single bird bag. The entrance hole to the nest box was blocked during the absence of the nestlings to prevent the return of the parents. Individual nestlings were removed from the bag one by one and held on their back in the palm of the hand to count the breath rate. The number of breaths was recorded for one minute, separating the number of breaths every 15 seconds (bout). After this each nestling was placed in a wooden box with individual compartments. After a 15-minute isolation period birds were removed from the box in the same order, and the frequency of breaths was again recorded for one minute (period 2). The bird was subsequently moved back into the bird bag. Once all the nestlings were tested, their tarsus and weight were measured after which nestlings were returned to their nest box. Raw means for the breath measurements and slopes are given in Table 3.

### Statistical analysis

To calculate an individual's estimate for the response to handling stress we used the same method as described in [49]. A linear mixed effect models (LMM) was used with the number of breaths per 15-second bout as dependent variable. Weight, temperature and temperature squared, date, tarsus, period (before or after social isolation) and bout (four 15-second bouts) were used as controlling variables. The actual individual responses to handling stress were derived by calculating the estimates from the interaction between individual and bout nested within period [for further details see 49]. This produces values that resemble the deviation from the slope in breath rates, and therefore provide a relative measure of handling stress, where higher levels indicate a steeper slope, i.e. a stronger response to handling.

We used a linear mixed effect model (LMM) along with nest of origin and nest of rearing nested within swap pair (two pairs of broods that were used to cross-foster) to assign variation in weight on day 2, day 8 and day 14 after hatching and the handling stress response to either genetic or rearing environment. For this we used REML estimation to estimate the random estimates and their standard errors. Swap pair was included as fixed factor to account for seasonal effects and variation in weight between pairs of broods in the analysis of weight on day two after hatching. An LMM was used to test the factors that influenced the amount of taurine delivered to the nest. We used the age of the chicks (four or eight days), the type of food (caterpillar or spider) as fixed factors and nest box as random factor. The proportion of spiders was arcsine square root–transformed to fit a normal distribution. Proportions between day four and day eight were compared using a paired t-test.

To test if food type and amount provisioned to the chicks influenced the handling stress response, we used an LMM with ML estimation with the handling stress estimate as a function of the biomass of spiders or caterpillars in the diet on day four and eight as fixed factors along with nest of origin and nest of rearing nested within swap pair as random factors. We performed a backwards elimination, removing the least significant term from the model until only terms that had P< 0.1 remained in the model. P values are given from the final model and from terms before they were eliminated from the model. To assess if our results could be explained by a weight difference between chicks, we added the weight at day 14 to our final model. All statistics were performed using SPSS versions 22 (IBM corp).

### Declarations

Publication costs for this article were funded by the German Research Foundation (FOR 1232) and the Open Access Publication Fund of Bielefeld and Muenster University.

### Competing interests

The authors declare that they have no competing interests.

### Authors’ contributions

KvO, GK and CH designed the research. GK and KvO performed the research. KvO and GK analysed the data. KvO and MN wrote the manuscript. All authors provided input during the writing of the manuscript.
